# Gene-Gene and Gene-Environment Interactions in Meta-Analysis of Genetic Association Studies

**DOI:** 10.1371/journal.pone.0124967

**Published:** 2015-04-29

**Authors:** Chin Lin, Chi-Ming Chu, John Lin, Hsin-Yi Yang, Sui-Lung Su

**Affiliations:** 1 Graduate Institute of Life Sciences, National Defense Medical Center, Taipei, Taiwan, ROC; 2 School of Public Health, National Defense Medical Center, Taipei, Taiwan, ROC; 3 Math Teachers’ Office, Kaohsiung Municipal Girls' Senior High School, Kaohsiung, Taiwan, ROC; Memorial Sloan Kettering Cancer Center, UNITED STATES

## Abstract

Extensive genetic studies have identified a large number of causal genetic variations in many human phenotypes; however, these could not completely explain heritability in complex diseases. Some researchers have proposed that the “missing heritability” may be attributable to gene–gene and gene–environment interactions. Because there are billions of potential interaction combinations, the statistical power of a single study is often ineffective in detecting these interactions. Meta-analysis is a common method of increasing detection power; however, accessing individual data could be difficult. This study presents a simple method that employs aggregated summary values from a “case” group to detect these specific interactions that based on rare disease and independence assumptions. However, these assumptions, particularly the rare disease assumption, may be violated in real situations; therefore, this study further investigated the robustness of our proposed method when it violates the assumptions. In conclusion, we observed that the rare disease assumption is relatively nonessential, whereas the independence assumption is an essential component. Because single nucleotide polymorphisms (SNPs) are often unrelated to environmental factors and SNPs on other chromosomes, researchers should use this method to investigate gene–gene and gene–environment interactions when they are unable to obtain detailed individual patient data.

## Introduction

Extensive genetic studies have identified a large number of causal genetic variations in many phenotypes; however, these could not completely explain the phenomenon of heritability in complex phenotypes [1]. Previous studies have suggested that the “missing heritability” may have been masked by gene–gene and gene–environment interactions, and therefore, their detection is very important. However, researchers have to carefully assess significance levels to reduce false discovery rates when determining the effect of interactions among multiple variables [2]; therefore, performing a single study is often ineffective under the correction of multiple comparisons [3,4].

Meta-analysis is a commonly used method for increasing detection power, and a subgroup analysis also could be used to detect the effects of interactions. Meta-analysis using individual patient data is considered the gold standard for investigating the moderator effect of participant-type variables [5,6]; however, access to detailed individual data could often be difficult. Meta-analyses using aggregate data have been more frequently employed because it maximizes the number of studies, patients, and events [7,8]. However, these methods are relatively difficult to apply in the meta-analysis of genetic association studies. It was difficult for researchers to obtain the population aggregated summary values of case-control studies; they often could only access the aggregated summary values in “cases” and “controls.” Unfortunately, most genetic association studies are designed as case-control investigations. Therefore, a method for detecting gene–gene and gene–environment interactions in a meta-analysis of a case-control study was imperative.

We hereby propose a simple method for detecting the effects of interactions in a meta-analysis of a case-control study. We have applied this method to an earlier study [9]. However, this was based on two assumptions (rare disease and independence), which may be violated in real data. The rare disease assumption is more frequently violated, and some researchers have debated on the extent of prevalence that should be established to classify the disease as “rare.” There is currently no evidence that could confirm the robustness of this method when the assumptions were violated. Therefore, this study aimed to test the 95% confidence interval coverage rate, power, and robustness of this method, and compare individual patient data analysis using simulation methods.

## Materials and Methods

### 2.1 Derivation of Formulas

Most genetic association studies utilize a case-control design, in which the association between the aggregated summary values of the factor and odd ratios was based on multiple factors. To better understand this principle, we hereby describe an example. In this example, the moderator can be not only factors of environment but also gene. That is a moderator implies any kind of covariates. This did not impact the results of derivation.

When the independent variable is an allele encoded with values of “minor allele” or “major allele” and the moderator is gender-encoded with values of “male” or “female,” the variables *E*_1_, *E*_2_, *E*_3_, and *E*_4_ in the population are the minor allele frequencies among the case women, case men, control women, and control men, respectively; these were based on seven population parameters, and their relationships are presented in the S1 Text. The odds ratio of exposure on disease outcome in women and men are as follows:

$$\text{Odds ratio}\;\left(\text{OR}\right)\;\text{in women}\;\left(OR_{women}\right):\;OR_{women}=\frac{E_{1}\left(1-E_{3}\right)}{E_{3}\left(1-E_{1}\right)}$$ Equation 2.1-1

$$\text{OR in men}\;\left(OR_{men}\right):\;OR_{men}=\frac{E_{2}\left(1-E_{4}\right)}{E_{4}\left(1-E_{2}\right)}$$ Equation 2.1-2

Based on these definitions, when a researcher conducts a case-control study, the expectation of a simple combined OR is affected by the proportion of males in the case group (*k*_1_) and control group (*k*_2_):

$$\text{Expectation of simple combined OR}\left(OR_{combine}\right):OR_{conbine}=\frac{\left(\left(1-k_{1}\right)E_{1}+k_{1}E_{2}\right)\left(\left(1-k_{2}\right)\left(1-E_{3}\right)+k_{2}\left(1-E_{4}\right)\right)}{\left(\left(1-k_{2}\right)E_{3}+k_{2}E_{4}\right)\left(\left(1-k_{1}\right)\left(1-E_{1}\right)+k_{1}\left(1-E_{2}\right)\right)}$$ Equation 2.1-3

The present study established the following two setting assumptions: (1) rare disease and (2) independence (there was no association between the factor of interest and the major independent variable), and *E*_3_ and *E*_4_ were similar to the proportion of individuals with exposure in the whole population; *p*_5_ was denoted the minor allele frequency in populations (S1 Text). Therefore, the *OR*_*combine*_ could be simplified as follows (*E*_3_ = *E*_4_ = *p*_5,_ please refer the S2 Text):

$$OR_{combine}=\frac{\left(\left(1-k_{1}\right)E_{1}+k_{1}E_{2}\right)\left(1-p_{5}\right)}{p_{5}\left(\left(1-k_{1}\right)\left(1-E_{1}\right)+k_{1}\left(1-E_{2}\right)\right)}$$ Equation 2.1-4

When moderator effects are present (*OR*_women_ ≠ *OR*_men_), the proportion of males in the case group (*k*_1_) is the only factor that could affect the *OR*_*combine*_. Researchers often perform a meta-regression to describe the association between the proportion of males and *OR*_*combine*_.

A typical single moderator equation of meta-regression (fixed-effect model) is shown in Eq 2.1-5 [The *y*_i_ is logarithmic empirical combined OR from each study [log(*OR*_*combine*_)], and we denote *η*_i_ as the residuals representing the unexplained errors of the reported *y*_i_] as follows:

$$y_{i}=b_{0}+b_{1}m_{i}+\eta_{i}$$ Equation 2.1-5

Where *m*_i_ is an unknown vector witch let Eq 2.1-5 holds. An appropriate *m*_i_ is calculated using Eq 2.1-6. When Eq 2.1-6 is used to access *m*_i_, *b*_0_ is considered to be the log(*OR*_*women*_), and *b*_1_ is considered the logarithmic moderator effect of gender [log(*OR*_*men*_) − log(*OR*_*women*_)] (The details of the derivation was shown in S3 Text).

$$m_{i}=\frac{\overset{\wedge }{y_{i}}-b_{0}}{b_{1}}=\frac{\text{log}\left[\left((1-k_{1i})E_{1}+k_{1i}E_{2})(1-E_{1}\right)\right]-\text{log}\left[((1-k_{1i})(1-E_{1})+k_{1i}(1-E_{2}))E_{1}\right]}{\text{log}\left[E_{2}(1-E_{1})\right]-\text{log}\left[E_{1}(1-E_{2})\right]}$$ Equation 2.1-6

Where *k*_1i_ is the summary value of case group in each study; *E*_1_, *E*_2_ are the minor allele frequencies of the respective case women and case men in each study. However, it was impossible to assess *m*_i_ because *E*_1_ and *E*_2_ were population parameters and most paper didn’t provide them. Fortunately, *m*_i_ is equal to *k*_1i_ when null hypothesis (null moderator effect) is satisfied (the details of theoretical proof was shown in the S4 Text). Therefore, we could use *k*_1i_ to replace *m*_i_ and create a new equation of meta-regression. The new equation of meta-regression is as follows:

$$y_{i}=b_{0}+b_{1}k_{1i}+\eta_{i}$$ Equation 2.1-7

Where, the *y*_i_, *k*_1i_, *η*_i_ are logarithmic empirical combined OR [log(*OR*_*combine*_)], the proportion of moderator in the “case” group, residuals representing the unexplained errors of the reported *y*_i_ from each study, respectively. Following above setting, the *b*_0_ and *b*_1_ are log(*OR*_*people without moderator*_), moderator effect, respectively. In this method, the coefficient of *b*_1_ can be represented the interaction between focus SNP and the moderator, such as gene-gene and gene-environment interactions. This could be employed to detect gene-gene interactions when *k*_1i_ is the minor allele frequency of another SNP and detect gene-environment interactions when *k*_1i_ is the proportion of environment exposure in the "case group."

Following Eq 2.1-7, the summary value of the case group (*k*_1i_) could be employed to build the meta-regression model, and the *b*_0_, *b*_1_ are log(*OR*_*people without moderator*_), moderator effect, respectively. The detailed calculated method of above coefficients and their variance were shown in S5 Text. In addition, S6 Text could help readers to understand the accuracy of Eq 2.1-7 when we violate the assumptions.

This could be employed to detect gene–gene interactions when *k*_1i_ is the minor allele frequency of another SNP and detect gene–environment interactions when *k*_1i_ is the proportion of environment exposure in the “case group.”

Individual patient data regression analysis is the gold standard in analyzing pooled data [6]. However, accessing the detailed trial results could be extremely difficult [7,8].

### 2.2 Simulations

Ten population parameters could be employed to describe the association between a disease, single nucleotide polymorphism (SNP), and moderator. The symbols *P*_1_, *P*_2_, *P*_3_, *P*_4_, *P*_5_, and *P*_6_ indicate the disease prevalence among people with homozygous major without moderator [*p*(*D* = 1|*x*_1_ = 0∩*x*_2_ = 0)], people with homozygous major with moderator [*p*(*D* = 1|*x*_1_ = 0∩*x*_2_ = 1)], people with heterozygous genotype without moderator [*p*(*D* = 1|*x*_1_ = 1∩*x*_2_ = 0)], people with heterozygous genotype with moderator [*p*(*D* = 1|*x*_1_ = 1∩*x*_2_ = 1)], people with homozygous minor without moderator [*p*(*D* = 1|*x*_1_ = 2∩*x*_2_ = 0)], and people with homozygous minor with moderator [*p*(*D* = 1|*x*_1_ = 2∩*x*_2_ = 1)], respectively. The symbol π_i_ denotes the minor allele frequency in each study population, and *P*_7_, *P*_8_, and *P*_9_ are the proportions of moderator status in people with homozygous major [*p*(*x*_2_ = 1|*x*_1_ = 0)], people with heterozygous genotype [*p*(*x*_2_ = 1|*x*_1_ = 1)], and people with homozygous minor [*p*(*x*_2_ = 1|*x*_1_ = 2)], respectively. *D* = disease status (0, health people; 1, patients). *x*_1_ = SNP (0, homozygous major; 1, heterozygous; 2, homozygous minor). *x*_2_ = moderator (0, without; 1, with).

It is worth noting that *x*_2_ can the genetic factor or environmental factor. The first step in generating simulation data is to set the parameters of the population. We assume that the moderator effect of the specific moderator is a fixed effect, and the association between SNP, the status of moderators, and the disease outcome in each study population is equal to following equation:

$$\text{log}(\frac{p}{1-p})=\beta_{0}+\beta_{1}x_{1}+\beta_{2}x_{2}+\beta_{3}x_{1}x_{2}$$ Equation 2.2-1

*p* = prevalence of outcome disease

In this equation, *β*_0_ is the logit-transformation prevalence of the outcome disease in people with homozygous major and without the moderators in study population. *β*_1_ is the log-transformation odds ratio of allele effect in people without moderators, *β*_2_ is the log-transformation OR of moderators on disease in people with homozygous major, and *β*_3_ is the log-transformation moderator effect. Following this model, we could set *β*_0_, *β*_1_, *β*_2_, and *β*_3_ to calculate *P*_1_, *P*_2_, *P*_3_, *P*_4_, *P*_5_, and *P*_6_. In our simulation, we set the mean of the minor allele frequency with 50% ($\bar{\pi }$), and we denote *F*_st_ as the frequency difference between different studies. The minor allele frequency (π_i_) in each study will be randomly generated from a beta distribution ($\alpha =\bar{\pi }\left(1-F_{st}\right)/F_{st}$; $\beta =\left(1-\bar{\pi }\right)\left(1-F_{st}\right)/F_{st}$), according to the Blading–Nichols model [10]. Under the Hardy–Weinberg equilibrium assumption, the frequency of homozygous major [*p*(*x*_1_ = 0)_i_, *q*_0i_], heterozygous [*p*(*x*_1_ = 1)_i_, *q*_1i_], and homozygous minor [*p*(*x*_1_ = 2)_i_, *q*_2i_] in each study were (1 − π_i_)^2^, 2π_i_(1 − π_i_), and π_i_^2^, respectively.

Table 1 summarizes the simulation conditions employed in the present study. There were five models (Basic, Minor violation of rare disease assumption, Serious violation of rare disease assumption, Minor violation of independence assumption, and Serious violation of independence assumption) in our simulation. We set the rare disease prevalence (10^−5^) in the Basic model; therefore, *β*_0_, the logit-transformation disease prevalence, is log[10^−5^/(1 − 10^−5^)]. Moreover, we set the odds ratios of allele effect and moderator effect as 1.5 and 2.0, respectively; therefore, *β*_1_ and *β*_2_ are log(1.5) and log(2.0), respectively. *P*_7_, *P*_8_, and *P*_9_ are the same in the Basic model and were set at 50%. Based on the Basic model, we set two kinds of models that violated the rare disease or independence assumptions, and there are two levels in each situation. The model Minor violation of rare disease assumption replaced *β*_0_ with log[10^−2^/(1 − 10^−2^)], and the model Serious violation of rare disease assumption replaced *β*_0_ with log[10^−1^/(1 − 10^−1^)]. The model Minor violation of independence assumption replaced *P*_7_, *P*_8_, and *P*_9_ with 0.4, 0.5, and 0.6, respectively, and the model Serious violation of rare disease assumption replaced *P*_7_, *P*_8_, and *P*_9_ with 0.3, 0.5, and 0.7, respectively.

**Table 1:** Summary of the population parameters.

Model	*β*_0_	*β*_1_	*β*_2_	*β*_3_	*F*_st_	*P*_7_	*P*_8_	*P*_9_
Basic	log[10^−5^/(1 − 10^−5^)]	log(1.5)	log(2)	0, 0.25, 0.5, 0.75, 1.0	0, 10^−2^, 10^−1^	0.5	0.5	0.5
Minor violation of rare disease assumption	log[10^−2^/(1 − 10^−2^)]	log(1.5)	log(2)	0, 0.25, 0.5, 0.75, 1.0	0, 10^−2^, 10^−1^	0.5	0.5	0.5
Serious violation of rare disease assumption	log[10^−1^/(1 − 10^−1^)]	log(1.5)	log(2)	0, 0.25, 0.5, 0.75, 1.0	0, 10^−2^, 10^−1^	0.5	0.5	0.5
Minor violation of independence assumption	log[10^−5^/(1 − 10^−5^)]	log(1.5)	log(2)	0, 0.25, 0.5, 0.75, 1.0	0, 10^−2^, 10^−1^	0.4	0.5	0.6
Serious violation of independence assumption	log[10^−5^/(1 − 10^−5^)]	log(1.5)	log(2)	0, 0.25, 0.5, 0.75, 1.0	0, 10^−2^, 10^−1^	0.3	0.5	0.7

*β*_0_ is the logit-transformation prevalence of the outcome disease in people with homozygous major and the moderators in the study population. *β*_1_ is the log-transformation OR of the allele effect in people without moderators. *β*_2_ is the log-transformation OR of moderators on the disease in people with homozygous major, and *β*_3_ is the log-transformation moderator effect. *F*_st_ is the frequency difference among various studies, and *P*_7_, *P*_8_, and *P*_9_ are the proportions of moderators status in people with homozygous major [*p*(*x*_2_ = 1|*x*_1_ = 0)], people with heterozygous genotype [*p*(*x*_2_ = 1|*x*_1_ = 1)], and people with homozygous minor [*p*(*x*_2_ = 1|*x*_1_ = 2)], respectively.

To conduct a meta-analysis of a genetic association study, we used the data from our past study [9] (S1 Table). In this data, the moderator (*x*_2_) was encoded with values the following values: people without moderator (*x*_2_ = 0) and with moderator (*x*_2_ = 1). There were 69 case-control studies that contained information regarding gender distribution as well as 14,692 cases and 13,414 controls. The genotype of each individual, which was encoded by values of 0, 1, or 2, was randomly generated from a multinomial distribution [*p* = *G*_1i_, *G*_2i_, *G*_3i_, and *G*_4i_, respectively]. *G*_1i_, *G*_2i_, *G*_3i_, and *G*_4i_ were the vector of genotype frequencies in cases without moderator [*p*(*x*_1_ = 0|*D* = 1∩*x*_2_ = 0)_i_, *p*(*x*_1_ = 1|*D* = 1∩*x*_2_ = 0)_i_, *p*(*x*_1_ = 2|*D* = 1∩*x*_2_ = 0)_i_], cases with moderator [*p*(*x*_1_ = 0|*D* = 1∩*x*_2_ = 1)_i_, *p*(*x*_1_ = 1|*D* = 1∩*x*_2_ = 1)_i_, *p*(*x*_1_ = 2|*D* = 1∩*x*_2_ = 1)_i_], controls without moderator [*p*(*x*_1_ = 0|*D* = 0∩*x*_2_ = 0)_i_, *p*(*x*_1_ = 1|*D* = 0∩*x*_2_ = 0)_i_, *p*(*x*_1_ = 2|*D* = 0∩*x*_2_ = 0)_i_], and controls with moderator [*p*(*x*_1_ = 0|*D* = 0∩*x*_2_ = 1)_i_, *p*(*x*_1_ = 1|*D* = 0∩*x*_2_ = 1)_i_, *p*(*x*_1_ = 2|*D* = 0∩*x*_2_ = 1)_i_], respectively. *G*_1i_, *G*_2i_, *G*_3i_, and *G*_4i_ were calculated based on *P*_1_, *P*_2_, *P*_3_, *P*_4_, *P*_5_, *P*_6_, *P*_7_, *P*_8_, *P*_9_, *q*_0i_, *q*_1i_, and *q*_2i_, respectively (S7 Text).

In the following analysis, we used our method to analyze the moderator effect using the summary data (Eq 2.1-7), and the summary odds ratio of each study was based on the additive model. The meta-regression used “metafor” packages [11] and the fixed-effect model was set to estimate the moderator effect. Moreover, the raw data were analyzed using individual patient data regression analysis. Individual patient data regression analysis was used for the hierarchical generalized linear model. Data in each condition were from 10,000 simulations.

The primary outcome was the 95% confidence interval coverage rate of the moderator effect (*β*_3_). The confidence interval coverage rate was the proportion of the 95% confidence interval, including the real parameter. The appropriate confidence interval coverage was 95%. In addition, type 1 errors were assessed in the null moderator effect model (*β*_3_ = 0). The secondary outcome was the power of moderator effect assessment because the nonsignificant result may often be ignored. In addition, researchers often reports the results of stratified analysis when the moderator effect was significance. We also presented the 95% confidence interval coverage rate of the allele effect in people without a moderator (*β*_1_) and people with a moderator (*β*_1_ + *β*_3_).

## Results

### Simulations under assumptions

Tables 2 and 3 present the results of the simulation. The Basic model is the simulation under the rare disease and independence assumptions. The 95% confidence interval coverage rates of our method were similar to the results of the individual patient data regression analysis regardless of condition and were close to 95%. Moreover, the false positive rates at a *p* = 0.05 significance threshold did not significantly differ from that observed using 5% in the null moderator effect model (*β*_3_ = 0). However, the power of our method was lower than the individual patient data regression analysis, indicating that the individual patient data regression analysis was more accurate. *F*st is the difference in allele frequencies among various studies. *F*st = 0, 0.01, and 0.1 indicated no differences, small difference, and large difference in allele frequency between the population and a specific ethnic group, respectively. The higher *F*st may reduce the power of the analysis, although this may not impact the stability of the 95% confidence interval coverage rates.

**Table 2:** 95% Confidence interval coverage rate, false positive rate, and power of moderator effect (%) at a 0.05 significance level using the present method.

Model	*F*_st_	*β*_3_ = 0	*β*_3_ = 0.25	*β*_3_ = 0.5	*β*_3_ = 0.75	*β*_3_ = 1.0
		CICR(FPR)	CICR(PWR)	CICR(PWR)	CICR(PWR)	CICR(PWR)
Basic	0	95.05(4.95)	95.40(17.01)	95.46(52.74)	95.23(84.83)	95.03(97.49)
	10^−2^	95.07(4.93)	95.25(17.43)	95.19(51.73)	95.41(85.01)	95.21(97.50)
	10^−1^	95.51(4.49)	95.25(16.58)	95.21(48.39)	95.15(82.39)	95.05(96.21)
Minor violation of rare disease assumption	0	94.84(5.16)	94.99(16.87)	95.10(49.71)	95.51(84.11)	95.72(97.48)
	10^−2^	94.99(5.01)	95.21(16.84)	95.30(50.31)	95.23(83.87)	95.16(97.04)
	10^−1^	94.94(5.06)	95.00(16.19)	94.92(47.32)	94.92(79.78)	95.21(95.54)
Serious violation of rare disease assumption	0	94.87(5.13)	94.51(14.23)	93.73(41.16)	92.98(73.97)	91.48(92.93)
	10^−2^	95.36(4.64)	94.61(13.73)	94.29(40.40)	93.57(73.54)	91.36(92.39)
	10^−1^	95.45(4.55)	94.85(12.71)	94.49(38.75)	93.59(70.46)	91.44(89.96)
Minor violation of independence assumption	0	91.04(8.96)	90.41(37.10)	90.61(73.98)	91.81(95.19)	91.45(99.46)
	10^−2^	90.99(9.01)	91.22(36.82)	90.50(74.53)	90.93(94.72)	91.28(99.40)
	10^−1^	91.56(8.44)	90.82(34.24)	91.29(50.42)	91.21(91.82)	91.83(98.67)
Serious violation of independence assumption	0	77.30(22.70)	76.92(60.50)	77.56(90.58)	78.96(98.91)	81.39(99.83)
	10^−2^	76.20(23.80)	77.45(60.12)	77.73(89.57)	78.99(98.52)	82.13(99.91)
	10^−1^	78.40(21.60)	78.88(56.90)	80.13(86.54)	80.58(97.61)	82.92(99.62)

CICR: 95% Confidence interval coverage rate of *β*_3_, including the real parameter; FPR: False positive rate; PWR: Statistical power, the proportion of significance.

**Table 3:** 95% Confidence interval coverage rate, false positive rate and power of moderator effect (%) at a 0.05 significance level in individual patient data regression analysis.

Model	*F*_st_	*β*_3_ = 0	*β*_3_ = 0.25	*β*_3_ = 0.5	*β*_3_ = 0.75	*β*_3_ = 1.0
		CICR(FPR)	CICR(PWR)	CICR(PWR)	CICR(PWR)	CICR(PWR)
Basic	0	95.10(4.90)	95.15(99.76)	95.13(100.00)	94.84(100.00)	95.23(100.00)
	10^−2^	95.03(4.97)	95.44(99.76)	95.12(100.00)	95.04(100.00)	95.45(100.00)
	10^−1^	95.26(4.74)	95.00(99.62)	95.47(100.00)	94.85(100.00)	95.59(100.00)
Minor violation of rare disease assumption	0	95.28(4.72)	95.44(99.81)	95.44(100.00)	94.80(100.00)	95.36(100.00)
	10^−2^	94.83(5.17)	95.14(99.75)	95.18(100.00)	95.54(100.00)	95.34(100.00)
	10^−1^	95.06(4.94)	95.62(99.66)	95.11(100.00)	95.48(100.00)	94.85(100.00)
Serious violation of rare disease assumption	0	95.10(4.90)	95.21(99.77)	95.03(100.00)	94.85(100.00)	95.25(100.00)
	10^−2^	95.42(4.58)	95.26(99.77)	95.20(100.00)	95.47(100.00)	95.40(100.00)
	10^−1^	95.42(4.58)	95.37(99.50)	95.18(100.00)	95.52(100.00)	95.33(100.00)
Minor violation of independence assumption	0	95.28(4.72)	95.39(99.72)	95.13(100.00)	94.80(100.00)	95.15(100.00)
	10^−2^	95.12(4.88)	95.03(99.75)	95.34(100.00)	95.52(100.00)	95.01(100.00)
	10^−1^	94.87(5.13)	95.25(99.52)	95.06(100.00)	95.11(100.00)	95.03(100.00)
Serious violation of independence assumption	0	95.24(4.76)	95.12(99.58)	95.36(100.00)	94.87(100.00)	94.69(100.00)
	10^−2^	95.64(4.36)	95.29(99.63)	95.23(100.00)	95.17(100.00)	94.64(100.00)
	10^−1^	95.00(5.00)	95.45(99.32)	95.28(100.00)	95.29(100.00)	94.97(100.00)

CICR: 95% Confidence interval coverage rate of *β*_3_, including the real parameter; FPR: False positive rate; PWR: Statistical power, the proportion of significance.

**Fig 1:**
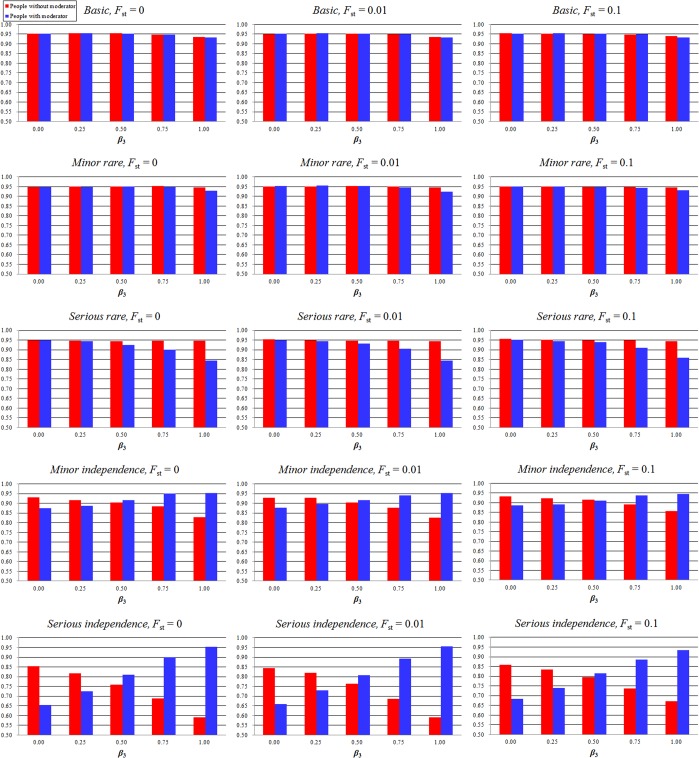
Confidence interval coverage rate of the allele effect in people without a moderator (*β*_1_) and people with a moderator (*β*_1_ + *β*_3_) using our proposed method. The model names, “Basic,” “Minor rare,” “Serious rare,” “Minor independence,” and “Serious independence” indicate the models, “Basic,” “Minor violation of rare disease assumption,” “Serious violation of rare disease assumption,” “Minor violation of independence assumption,” and “Serious violation of independence assumption,” respectively. *F*_st_ is the parameter of frequency difference among various studies. The X-axis represents the confidence interval of the moderator effect (*β*_3_); the Y-axis represents the 95% confidence interval coverage rate. The red bar represents the 95% confidence interval coverage rate of the allele effect in people without a moderator (*β*_1_); the blue bar represents the 95% confidence interval coverage rate of the allele effect in people with a moderator (*β*_1_ + *β*_3_).

**Fig 2:**
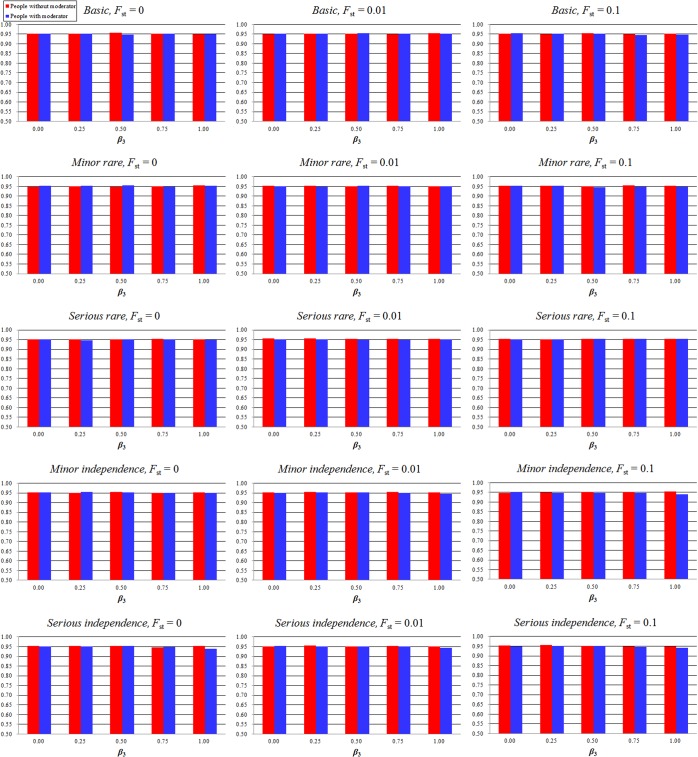
Confidence interval coverage rate of the allele effect in people without a moderator (*β*_1_) and people with a moderator (*β*_1_ + *β*_3_) in individual patient data regression analysis. The model names, “Basic,” “Minor rare,” “Serious rare,” “Minor independence,” and “Serious independence” indicate the models, “Basic,” “Minor violation of rare disease assumption,” “Serious violation of rare disease assumption,” “Minor violation of independence assumption,” and “Serious violation of independence assumption,” respectively. *F*_st_ is the parameter of frequency difference among various studies. The X-axis represents the confidence interval of the moderator effect (*β*_3_); the Y-axis represents the 95% confidence interval coverage rate. The red bar represents the 95% confidence interval coverage rate of the allele effect in people without a moderator (*β*_1_); the blue bar represents the 95% confidence interval coverage rate of the allele effect in people with a moderator (*β*_1_ + *β*_3_).

Figs 1 and 2 show the 95% confidence interval coverage rate of the allele effect in people without a moderator (*β*_1_) and people with a moderator (*β*_1_ + *β*_3_). The 95% confidence interval coverage rates of our method were close to 95% in any condition under the rare disease and independence assumptions. The individual patient data regression analysis was also robust in this situation.

### Simulations with violations of assumptions

Two models, Minor violation of rare disease assumption and Serious violation of rare disease assumption, tested the robustness when the outcome disease is not a rare disease, and we set the 1% and 10% disease prevalence rates in people with homozygous major without moderator, respectively. In the null moderator effect model analysis, the false positive rate of our method did not significantly differ from the 5% in any model and *F*st. However, we observed that the 95% confidence interval coverage rates of our method were lower in the higher moderator effect model (*β*_3_ = 0.25–1.0). The extent of reduction was impacted by disease prevalence and moderator effect; the simulation with higher disease prevalence and moderator effect showed lower 95% confidence interval coverage rates. Moreover, the analytical power was reduced because of higher disease prevalence. The individual patient data regression analysis remained robust regardless of the condition. The 95% confidence interval coverage rate of the allele effect in people without a moderator and people with a moderator was also lower in the Serious violation of rare disease assumption model, and the extent of reduction was impacted by the moderator effect.

We tested the robustness of our method when the situation violated the independence assumption. We set the small difference (0.1) and large difference (0.2) between *P*_7,_ *P*_8_, and *P*_9_, which indicated a small and strong association between SNP and moderator. We observed that the 95% confidence interval coverage rates of our method were lower in the model with violation of independence assumptions, and the extent of reduction was impacted by the strength of association between SNP and moderator. Moreover, the false positive rates of our method were significantly different from 5%. Therefore, the power analysis in this scenario was insignificant. The association between SNP and moderator did not impact the robustness of individual patient data regression analysis. Its 95% confidence interval coverage rates remained close to 95%, and it had appropriate false positive rates and high powers in any condition. Similar to the moderator effect, the results of allele effect in people without a moderator and people with a moderator showed that our method was not robust in the model with the violation of independence assumptions. The individual patient data regression analysis was also robust in any situation.

## Discussion

This work is trying to propose a new method for meta-analysis when researchers were unable to obtain the raw data of each individual sample. It is difficult for accessing the detailed individual data [5,6]. Meta-analyses using aggregate data have been more frequently employed because it maximizes the number of studies, patients, and events [7,8]. However, there is no suitable methods for case-control studies but most genetic association studies are designed as case-control investigations. We believe this approach is an alternative to investigate more information of gene-gene and gene-environment interactions.

Previous papers generally present the stratified results of minority participant types such as smoking status, and researchers utilize such information to assess their moderator effects [12]. However, most participant-type variables, such as other SNPs and gender, are presented as average summary values. Several meta-analyses of case-control studies consider that the absence of a control for various participant types was an important limitation and the exposure to different environmental factors could be difficult to completely assess [13–16]. The use of the meta-regression model using summary values has been employed for years. Some previous studies have used the summary values of the case group to determine the source of heterogeneity [17,18], whereas others have used the summary value of the control group [19]. One study even used the summary values of both the case and control groups [20]. However, these studies did not describe their bases for their selection of the summary value of a specific study group. Moreover, they often did not explain the biological significance of their analysis. The present study evaluated the biological significance of using the summary value of the case group in assessing their moderator effects, particularly when individual patient data could not be collected.

Individual patient data analysis had the higher confidence interval coverage rate and power, and this result was similar to that of previous simulation studies on meta-analyses of RCT [6]. However, accessing the detailed trial results can be difficult [7,8]. The standard error of individual patient data analysis was smaller than the standard error of our method, implying that the estimates of individual patient data analysis were more accurate. Therefore, we recommend that researchers contact the authors of included reports to obtain more detailed data and use our method as a last resort when they are unable to obtain sufficient information.

The independence assumption is important because the relationship between summary values and odds ratios does not follow a linear correlation when it occurs as a Simpson’s paradox. The independence assumption could avoid the Simpson’s paradox to determine whether the robustness of our method was insufficient when the situation violated the independence assumption. The rare disease assumption was relatively unimportant because the association between the summary values and odds ratios continued to follow a linear correlation. Therefore, the false positive rate did not increase when the situation violated the rare disease assumption. However, with the increase in disease prevalence, the effect of the summary value from the “case” and “control” on odds ratio changed. When the actual disease prevalence approached 0%, the summary value from the “case” was the only factor that influenced the estimator of the combined odds ratio. When the true disease prevalence approached 100%, the summary value from the “control” was the only factor that influenced the estimator of the combined odds ratio. In fact, the impact of the summary value from the “case” and “control” were based on the actual disease prevalence. However, diseases with >50% prevalence rates may not be present; therefore, we considered that the impact of the summary value from the “case” was always larger than the summary value from the “control.” Because researchers often could not obtain actual disease prevalence rates, we considered that detecting the interactions using the summary value from a “case” was a suitable selection. In fact, the results of the meta-regression using the summary value from the case and control groups were similar because most of the studies had similar proportions of moderators in the case or control groups (e.g., matched studies). However, using the summary value of a case group was apparently a better selection because the impact weight of the summary value from the “case” was higher than that of the summary value from the “control” unless the real disease prevalence was >50%.

In conclusion, we considered that building the meta-regression using the summary value from a case group may be an effective approach when the information from every individual patient in insufficient. Furthermore, this approach is extremely easy to use and could assist in defining the biological significance. Several software programs can conduct meta-regression analysis such as R and STATA, and researchers can use these to investigate the interaction between the factor of interest, such as other SNPs or environment factor, and topic SNP. On the other hand, the rare disease assumption is relatively unimportant. However, when the actual disease prevalence is >10%, the estimators of meta-regression could be distorted, although the significant interactions may still possibly remain true. The independence assumption is important. The detection method for this interaction may largely deviate from the real situation, particularly when this violates the independence assumption. However, SNPs are often unrelated to environmental factors and SNPs of other chromosomes. Therefore, these results indicate that this method is useful in genetic studies. The meta-analysis of genetic association studies could also be effectively used in detecting gene–gene and gene–environment interactions, which may be accountable for the “missing heritability.”

## Supporting Information

S1 Text The relationship between population parameters and the minor allele frequencies. (DOCX)

S2 Text Details of the derivation of Eq 2.1-3 and Eq 2.1-4. (DOCX)

S3 Text Details of the derivation of Eq 2.1-6. (DOCX)

S4 Text The theoretical proof of Eq 2.1-5 to Eq 2.1-7. (DOCX)

S5 Text The detailed calculated method of Eq 2.1-7. (DOCX)

S6 Text A simple way to understand the Eq 2.1-7 and two assumptions. (DOCX)

S7 Text The relationship between *G*_1_, *G*_2_, *G*_3_, *G*_4_ and *P*_1_, *P*_2_, *P*_3_, *P*_4_, *P*_5_, *P*_6_, P_7_, *P*_8_, *P*_9_, q_0_, *q*_1_, *q*_2_. (DOCX)

S1 Table Detailed data in the real dataset. (DOCX)
