# Supplementary material for: Gene-Gene and Gene-Environment Interactions in Meta-Analysis of Genetic Association Studies
Source: PLoS One. 2015 Apr 29;10(4):e0124967. doi: 10.1371/journal.pone.0124967 (PMC4414456; doi:10.1371/journal.pone.0124967)
Supplement: S1 Text — (DOCX) [file pone.0124967.s001.docx]

**The relationship between population parameters and the minor allele frequencies**

When the single nucleotide polymorphism, with values of ‘minor allele’ or ‘major allele’ and the moderator is gender, with values of ‘male’ or ‘female’, the variables *p*_1_, *p*_2_, *p*_3_ and *p*_4_ are the outcome prevalence of women with major allele, men with major allele, women with minor allele and men with minor allele. The variable *p*_5_ is the minor allele frequency in the whole population, *p*_6_ is the proportion of men in the population with major allele and *p*_7_ is the proportion of men in the population with minor allele. The minor allele frequency among case women (*E*_1_), case men (*E*_2_), control women (*E*_3_) and control men (*E*_4_) were presented as follows:

Minor allele frequency among case women (*E*_1_):$E_{1}=\frac{p_{3}p_{5}\left( 1-p_{7} \right)}{p_{1}\left( 1-p_{5} \right)\left( 1-p_{6} \right)+p_{3}p_{5}\left( 1-p_{7} \right)}$

Minor allele frequency among case men (*E*_2_):$\log\left( \frac{A_{2}\times D_{2}}{B_{2}\times C_{2}} \right)$

Minor allele frequency among control women (*E*_3_):$E_{3}=\frac{\left( 1-p_{3} \right)p_{5}\left( 1-p_{7} \right)}{\left( 1-p_{1} \right)\left( 1-p_{5} \right)\left( 1-p_{6} \right)+\left( 1-p_{3} \right)p_{5}\left( 1-p_{7} \right)}$

Minor allele frequency among control men (*E*_4_): $E_{4}=\frac{\left(1-p_{4}\right)p_{5}p_{7}}{\left(1-p_{2}\right)\left(1-p_{5}\right)p_{6}+\left(1-p_{4}\right)p_{5}p_{7}}$
