# Supplementary material for: Gene-Gene and Gene-Environment Interactions in Meta-Analysis of Genetic Association Studies
Source: PLoS One. 2015 Apr 29;10(4):e0124967. doi: 10.1371/journal.pone.0124967 (PMC4414456; doi:10.1371/journal.pone.0124967)
Supplement: S2 Text — (DOCX) [file pone.0124967.s002.docx]

**Details of the derivation of Equation 2.1-3 to Equation 2.1-4:**

There are two setting assumptions to simplify E_3_ and E_4_: (1) rare disease (*p*_1_, *p*_2_, *p*_3_, *p*_4_ are very rare) and (2) independence (*p*_6_ = *p*_7_):

$$\begin{matrix} E_{3}=\lim_{p_{1}\to0} \lim_{p_{3}\to0} \left(\frac{\left(1-p_{3}\right)p_{5}\left(1-p_{7}\right)}{\left(1-p_{1}\right)\left(1-p_{5}\right)\left(1-p_{6}\right)+\left(1-p_{3}\right)p_{5}\left(1-p_{7}\right)}\right) \\ =\frac{p_{5}\left(1-p_{7}\right)}{\left(1-p_{5}\right)\left(1-p_{6}\right)+p_{5}\left(1-p_{7}\right)} \\ =\frac{p_{5}\left(1-p_{6}\right)}{\left(1-p_{5}\right)\left(1-p_{6}\right)+p_{5}\left(1-p_{6}\right)}\text{ "}※p_{6}=p_{7}\text{"} \\ =p_{5} \end{matrix}$$

$$\begin{matrix} E_{4}=\lim_{p_{2}\to0} \lim_{p_{4}\to0} \left(\frac{\left(1-p_{4}\right)p_{5}p_{7}}{\left(1-p_{2}\right)\left(1-p_{5}\right)p_{6}+\left(1-p_{4}\right)p_{5}p_{7}}\right) \\ =\frac{p_{5}p_{7}}{\left(1-p_{5}\right)p_{6}+p_{5}p_{7}} \\ =\frac{p_{5}p_{6}}{\left(1-p_{5}\right)p_{6}+p_{5}p_{6}}\text{ "}※p_{6}=p_{7}\text{"} \\ =p_{5} \end{matrix}$$

Therefore, the Equation 2.1-3 can be simplified to the Equation 2.1-4:

$$\begin{matrix} OR_{conbine}=\frac{\left(\left(1-k_{1}\right)E_{1}+k_{1}E_{2}\right)\left(\left(1-k_{2}\right)\left(1-E_{3}\right)+k_{2}\left(1-E_{4}\right)\right)}{\left(\left(1-k_{2}\right)E_{3}+k_{2}E_{4}\right)\left(\left(1-k_{1}\right)\left(1-E_{1}\right)+k_{1}\left(1-E_{2}\right)\right)} \\ =\frac{\left(\left(1-k_{1}\right)E_{1}+k_{1}E_{2}\right)\left(\left(1-k_{2}\right)\left(1-p_{5}\right)+k_{2}\left(1-p_{5}\right)\right)}{\left(\left(1-k_{2}\right)p_{5}+k_{2}p_{5}\right)\left(\left(1-k_{1}\right)\left(1-E_{1}\right)+k_{1}\left(1-E_{2}\right)\right)} \\ =\frac{\left(\left(1-k_{1}\right)E_{1}+k_{1}E_{2}\right)\left(1-p_{5}\right)}{p_{5}\left(\left(1-k_{1}\right)\left(1-E_{1}\right)+k_{1}\left(1-E_{2}\right)\right)} \end{matrix}$$
