# Supplementary material for: Gene-Gene and Gene-Environment Interactions in Meta-Analysis of Genetic Association Studies
Source: PLoS One. 2015 Apr 29;10(4):e0124967. doi: 10.1371/journal.pone.0124967 (PMC4414456; doi:10.1371/journal.pone.0124967)
Supplement: S3 Text — (DOCX) [file pone.0124967.s003.docx]

**Details of the derivation of Equation 2.1-6:**

The *b*_0_ is considered to be the log(OR_women_), and *b*_1_ is considered the logarithmic moderator effect of gender [log(OR_men_) − log(OR_women_)]. The *y*_i_ is logarithmic empirical combined OR from each study [log(*OR_combine_*)].

$$b_{0}=\log(OR_{women})=\log\left(\frac{E_{1}\left(1-E_{3}\right)}{E_{3}\left(1-E_{1}\right)}\right)=\log\left(\frac{E_{1}\left(1-p_{5}\right)}{p_{5}\left(1-E_{1}\right)}\right)$$

$$\begin{matrix} b_{1}=\log(OR_{men})-\log(OR_{women})= \\ \log\left(\frac{E_{2}\left(1-E_{4}\right)}{E_{4}\left(1-E_{2}\right)}\right)-\log\left(\frac{E_{1}\left(1-E_{3}\right)}{E_{3}\left(1-E_{1}\right)}\right)=\log\left(\frac{E_{2}\left(1-p_{5}\right)}{p_{5}\left(1-E_{2}\right)}\right)-\log\left(\frac{E_{1}\left(1-p_{5}\right)}{p_{5}\left(1-E_{1}\right)}\right)=\log\left(\frac{E_{2}\left(1-E_{1}\right)}{E_{1}\left(1-E_{2}\right)}\right) \end{matrix}$$

$\mathop{y_{i}}\limits^{\wedge} =\log(OR_{combine})=\log\left(\frac{\left(\left(1-k_{1}\right)E_{1}+k_{1}E_{2}\right)\left(1-p_{5}\right)}{p_{5}\left(\left(1-k_{1}\right)\left(1-E_{1}\right)+k_{1}\left(1-E_{2}\right)\right)}\right)$The detailed of the derivation was shown as follows:

$$\begin{matrix} m_{i}=\frac{\mathop{y_{i}}\limits^{\wedge} -b_{0}}{b_{1}}=\frac{\log\left(\frac{\left(\left(1-k_{1}\right)E_{1}+k_{1}E_{2}\right)\left(1-p_{5}\right)}{p_{5}\left(\left(1-k_{1}\right)\left(1-E_{1}\right)+k_{1}\left(1-E_{2}\right)\right)}\right)-\log\left(\frac{E_{1}\left(1-p_{5}\right)}{p_{5}\left(1-E_{1}\right)}\right)}{\log\left(\frac{E_{2}\left(1-E_{1}\right)}{E_{1}\left(1-E_{2}\right)}\right)} \\ =\frac{\log\left(\left(1-k_{1}\right)E_{1}+k_{1}E_{2}\right)\left(1-p_{5}\right)-\log p_{5}\left(\left(1-k_{1}\right)\left(1-E_{1}\right)+k_{1}\left(1-E_{2}\right)\right)-\log E_{1}\left(1-p_{5}\right)+\log p_{5}\left(1-E_{1}\right)}{\log E_{2}(1-E_{1})-\log E_{1}(1-E_{2})} \\ =\frac{\log\left(\left(1-k_{1}\right)E_{1}+k_{1}E_{2}\right)+\log\left(1-p_{5}\right)-\log p_{5}-\log\left(\left(1-k_{1}\right)\left(1-E_{1}\right)+k_{1}\left(1-E_{2}\right)\right)-\log E_{1}-\log\left(1-p_{5}\right)+\log p_{5}+\log\left(1-E_{1}\right)}{\log E_{2}(1-E_{1})-\log E_{1}(1-E_{2})} \\ =\frac{\log\left(\left(1-k_{1}\right)E_{1}+k_{1}E_{2}\right)-\log\left(\left(1-k_{1}\right)\left(1-E_{1}\right)+k_{1}\left(1-E_{2}\right)\right)-\log E_{1}+\log\left(1-E_{1}\right)}{\log\left[E_{2}(1-E_{1})\right]-\log\left[E_{1}(1-E_{2})\right]} \\ =\frac{\log\left[((1-k_{1i})E_{1}+k_{1i}E_{2})(1-E_{1})\right]-\log\left[((1-k_{1i})(1-E_{1})+k_{1i}(1-E_{2}))E_{1}\right]}{\log\left[E_{2}(1-E_{1})\right]-\log\left[E_{1}(1-E_{2})\right]} \end{matrix}$$
