# Supplementary material for: Gene-Gene and Gene-Environment Interactions in Meta-Analysis of Genetic Association Studies
Source: PLoS One. 2015 Apr 29;10(4):e0124967. doi: 10.1371/journal.pone.0124967 (PMC4414456; doi:10.1371/journal.pone.0124967)
Supplement: S4 Text — (DOCX) [file pone.0124967.s004.docx]

**The theoretical proof of Equation 2.1-5 to Equation 2.1-7:**

**Equation 2.1-5:** $y_{i}=b_{0}+b_{1}m_{i}+\eta_{i}$

Where the *b*_0_ is considered to be the log(OR_women_), and *b*_1_ is considered the logarithmic moderator effect of gender [log(OR_men_) − log(OR_women_)]. The *y*_i_ is logarithmic empirical combined OR from each study [log(*OR_combine_*)]. The *m*_i_ is an unknown vector witch let Equation 2.1-5 holds, and a suitable *m*_i_ can be calculated by Equation 2.1-6 as follows:

**Equation 2.1-6:** $m_{i}=\frac{\mathop{y_{i}}\limits^{\wedge} -b_{0}}{b_{1}}=\frac{\log\left[((1-k_{1i})E_{1}+k_{1i}E_{2})(1-E_{1})\right]-\log\left[((1-k_{1i})(1-E_{1})+k_{1i}(1-E_{2}))E_{1}\right]}{\log\left[E_{2}(1-E_{1})-\log E_{1}(1-E_{2})\right]}$

However, it was impossible to assess *m*_i_ because *E*_1_ and *E*_2_ were population parameters and most paper didn’t provide them. Fortunately, *m* _i_ is equal to *k*_1i_ when null hypothesis (null moderator effect) is satisfied. The theoretical proof was shown as follows:

The null hypothesis is null moderator effect (*b*_1_ = 0), and the equation of was shown as follows:

$$b_{1}=\log\left(\frac{E_{2}\left(1-E_{1}\right)}{E_{1}\left(1-E_{2}\right)}\right)=\log\left(\frac{(E_{1}+\varepsilon)\left(1-E_{1}\right)}{E_{1}\left(1-E_{1}-\varepsilon\right)}\right)$$

Where *E*_1_, *E*_2_ are the minor allele frequency among case women, case men, respectively. We used *E*_1_+ε to replace the *E*_2_, and the only situation of null moderator effect (*b*_1_ = 0) is that the ε is equal to 0. Above change can change the equation 2.1-6 as follows:

$$\begin{matrix} m_{i}=\frac{\log\left[((1-k_{1i})E_{1}+k_{1i}E_{2})(1-E_{1})\right]-\log\left[((1-k_{1i})(1-E_{1})+k_{1i}(1-E_{2}))E_{1}\right]}{\log\left[E_{2}(1-E_{1})\right]-\log\left[E_{1}(1-E_{2})\right]} \\ =\frac{\log\left[((1-k_{1i})E_{1}+k_{1i}(E_{1}+\varepsilon))(1-E_{1})\right]-\log\left[((1-k_{1i})(1-E_{1})+k_{1i}(1-(E_{1}+\varepsilon)))E_{1}\right]}{\log\left[(E_{1}+\varepsilon)(1-E_{1})\right]-\log\left[E_{1}(1-(E_{1}+\varepsilon))\right]} \\ =\frac{\log\left[(E_{1}+k_{1i}\varepsilon)(1-E_{1})\right]-\log\left[E_{1}(1-(E_{1}+k_{1i}\varepsilon))\right]}{\log\left[(E_{1}+\varepsilon)(1-E_{1})\right]-\log\left[E_{1}(1-(E_{1}+\varepsilon))\right]} \end{matrix}$$

Therefore, the *m* _i_ is equal to *k*_1i_ when the ε is close to 0 as follows:

Based on above proof, we could use *k*_1i_ to replace *m*_i_ in Equation 2.1-5 and create a new equation of meta-regression. The Equation 2.1-7 of meta-regression is as follows:

**Equation 2.1-7:** $y_{i}=b_{0}+b_{1}k_{1i}+\eta_{i}$
