# Supplementary material for: Gene-Gene and Gene-Environment Interactions in Meta-Analysis of Genetic Association Studies
Source: PLoS One. 2015 Apr 29;10(4):e0124967. doi: 10.1371/journal.pone.0124967 (PMC4414456; doi:10.1371/journal.pone.0124967)
Supplement: S5 Text — (DOCX) [file pone.0124967.s005.docx]

**The detailed calculated method of Equation 2.1-7:**

**Equation 2.1-7:** $y_{i}=b_{0}+b_{1}k_{1i}+\eta_{i}$

Equation 2.1-7 is the final model for detecting the moderator effect, where the *y*_i_, *k*_1i_, *η*_i_ are logarithmic empirical combined OR [log(*OR_combine_*)], the proportion of moderator in the case group, residuals representing the unexplained errors of the reported *y*_i_ from each study, respectively. Our objective is that calculate and test *b*_0_[log(*OR_people without moderator_*)], and *b*_1_[moderator effect, log(*OR_people with moderator_*) − log(*OR_people without moderator_*)]. In fixed effect model, the Equation 2.1-7 can be expressed as follows:

$$Y=XB+E$$

Where:

*Y* is the N by 1 vector of all the logarithmic reported OR from each study.

*X* is the N by M matrix of moderator variables (the moderator variable is *k*_1i_ in Equation 2.1-7), the first column of which contains all 1s.

*B* is the M by 1 vector of meta-regression coefficients, the first one is intercept.

*E* is the N by 1 vector of all the residuals representing the unexplained errors of the reported *y*_i_ from each study.

$Y=\left[\begin{matrix} y_{1} \\ y_{2} \\ ... \\ y_{N} \end{matrix}\right]$ $X=\left[\begin{matrix} 1 & x_{1,1} & x_{1,2} & ... & x_{1,M-1} \\ 1 & x_{2,1} & x_{2,2} & ... & x_{2,M-1} \\ ... & ... & ... & ... & ... \\ 1 & x_{N,1} & x_{N,2} & ... & x_{N,M-1} \end{matrix}\right]$ $B=\left[\begin{matrix} b_{0} \\ b_{1} \\ b_{2} \\ ... \\ b_{M-1} \end{matrix}\right]$ $E=\left[\begin{matrix} \varepsilon_{1} \\ \varepsilon_{2} \\ ... \\ \varepsilon_{N} \end{matrix}\right]$

In addition, the weights of each study are based on their variance of logarithmic reported OR (σ^2^), and the matrix W is shown as follows:

$$W=\left[\begin{matrix} \frac{1}{\sigma_{1}^{2}} & 0 & ... & 0 \\ 0 & \frac{1}{\sigma_{2}^{2}} & ... & 0 \\ ... & ... & ... & ... \\ 0 & 0 & ... & \frac{1}{\sigma_{N}^{2}} \end{matrix}\right]$$

The vector *B* can be calculated by following equation:

$$B=\left(X^{T}WX\right)^{-1}X^{T}WY$$

And the variance covariance matrix of *B* can be calculated by follows:

$$\mathrm{var}\left(B\right)=\left(X^{T}WX\right)^{-1}$$

Therefore, the *b*_0_, *b*_1_ and their variance are shown as follows:

$b_{0}=B_{1,1}$; $\mathrm{var}\left(b_{0}\right)=\mathrm{var}\left(B\right)_{1,1}$

$b_{1}=B_{2,1}$; $\mathrm{var}\left(b_{1}\right)=\mathrm{var}\left(B\right)_{2,2}$
