# Supplementary material for: Gene-Gene and Gene-Environment Interactions in Meta-Analysis of Genetic Association Studies
Source: PLoS One. 2015 Apr 29;10(4):e0124967. doi: 10.1371/journal.pone.0124967 (PMC4414456; doi:10.1371/journal.pone.0124967)
Supplement: S6 Text — (DOCX) [file pone.0124967.s006.docx]

**A simple way to understand the Equation 2.1-7 and two assumptions:**

**Equation 2.1-7:** $y_{i}=b_{0}+b_{1}k_{1i}+\eta_{i}$

Equation 2.1-7 is the final model for detecting the moderator effect, where the *k*_1i_ and *y*_i_ is summary value of the case group and logarithmic empirical combined OR in each study, respectively. However, this model was based on two assumptions: (1) rare disease and (2) independence. We developed a website (<http://geogebratube.com/student/mabDuCWqx>) to help readers to understand the bias when we violate the assumptions.

This website is an example for investigating the moderate effect of gender on the association between exposure and outcome disease. The p_1_ to p_7_ are the population parameters (the definitions and relationship between them and *E*_1_, *E*_2_, *E*_3_, *E*_4_ were shown in the start of supplementary file), and the odds ratios in women (ORw) and in men (ORm) are calculated based on Equation 2.1-1 and Equation 2.1-2.

**Equation 2.1-1:** Odds ratio (OR) in women (*OR_women_*): $\mathrm{OR}_{\mathrm{women}}=\frac{E_{1}\times\left( 1-E_{3} \right)}{E_{3}\times\left( 1-E_{1} \right)}$

**Equation 2.1-2:** OR in men (*OR_men_*): $\mathrm{OR}_{\mathrm{men}}=\frac{E_{2}\times\left( 1-E_{4} \right)}{E_{4}\times\left( 1-E_{2} \right)}$

If a researcher want to do a case control study, the expectation of simple combined OR [OR(combined), ORc] is calculated based on Equation 2.1-3, and it is based on by proportion of males in the case group (k1) and in the control group (k2).

**Equation 2.1-3:** Expectation of simple combined OR (*OR_combine_*): $\mathrm{OR}_{\mathrm{crude}}=\frac{\left( {\left( 1-k_{1} \right)E}_{1}+{k_{1}E}_{2} \right)\times\left( \left( 1-k_{2} \right)E_{3}+{k_{2}E}_{4} \right)}{\left( \left( 1-k_{1} \right)\left( 1-E_{1} \right)+k_{1}\left( 1-E_{2} \right) \right)\times\left( \left( 1-k_{2} \right)\left( 1-E_{3} \right)+k_{2}\left( 1-E_{4} \right) \right)}$

The ORλ is calculated based on the Equation 2.1-7 (please refer the start of this part). The main focus of this webpage is to observe the difference between log(ORc) and log(ORλ),


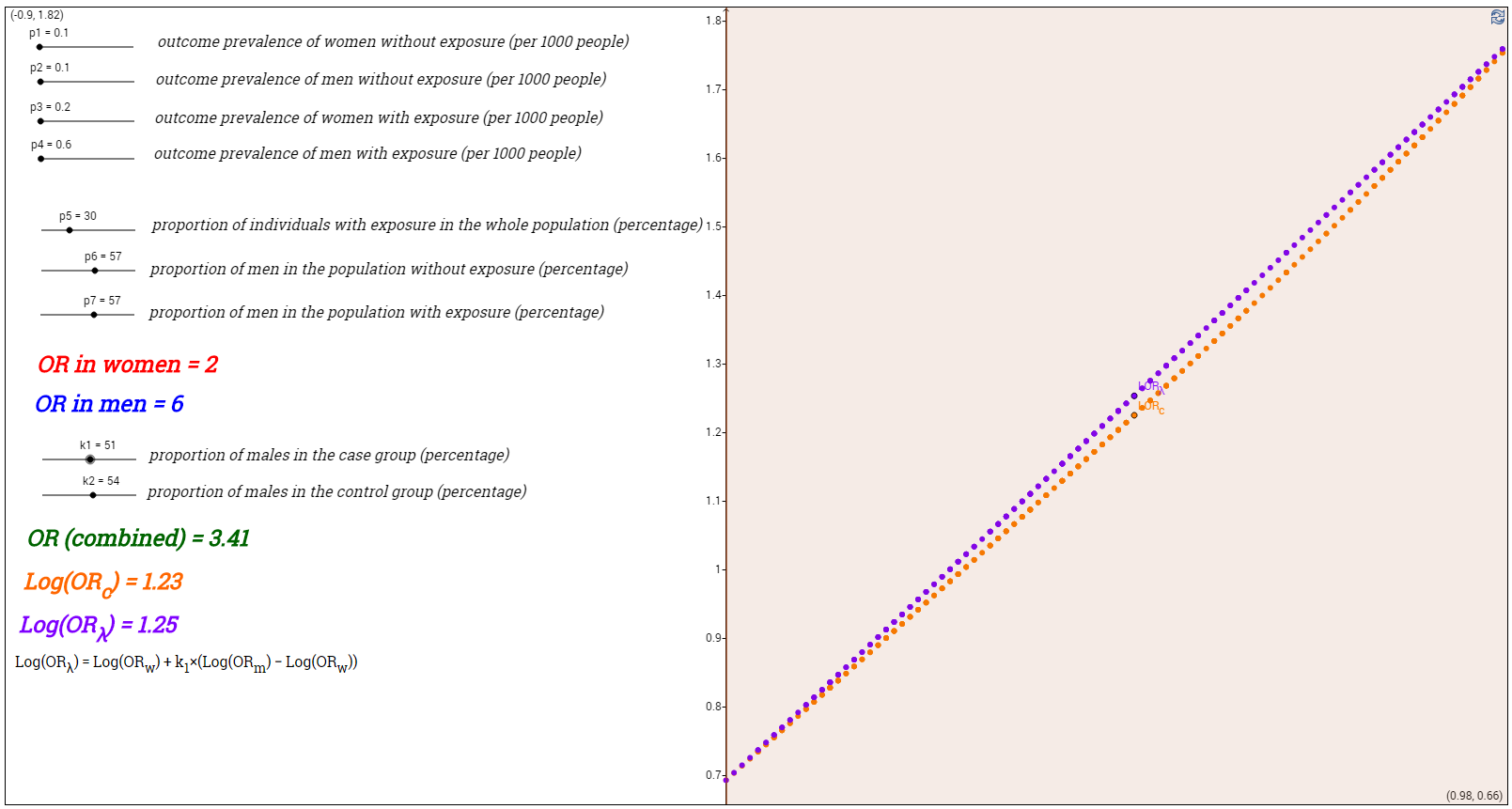


**Figure S1 The screenshot of website under two assumptions**

The x-axis is the proportion of males in the case group (k1), and the y-axis is the log of odds ratios [including log(ORc) (orange) and log(ORλ)) (purple)].

We could observe the orange trace is close to purple trace when *p*_1_, *p*_2_, *p*_3_ and *p*_4_ are rare, so we could understand the log(ORc) and log(ORλ) will be very close under two assumptions: (1) rare disease, and (2) independence.:


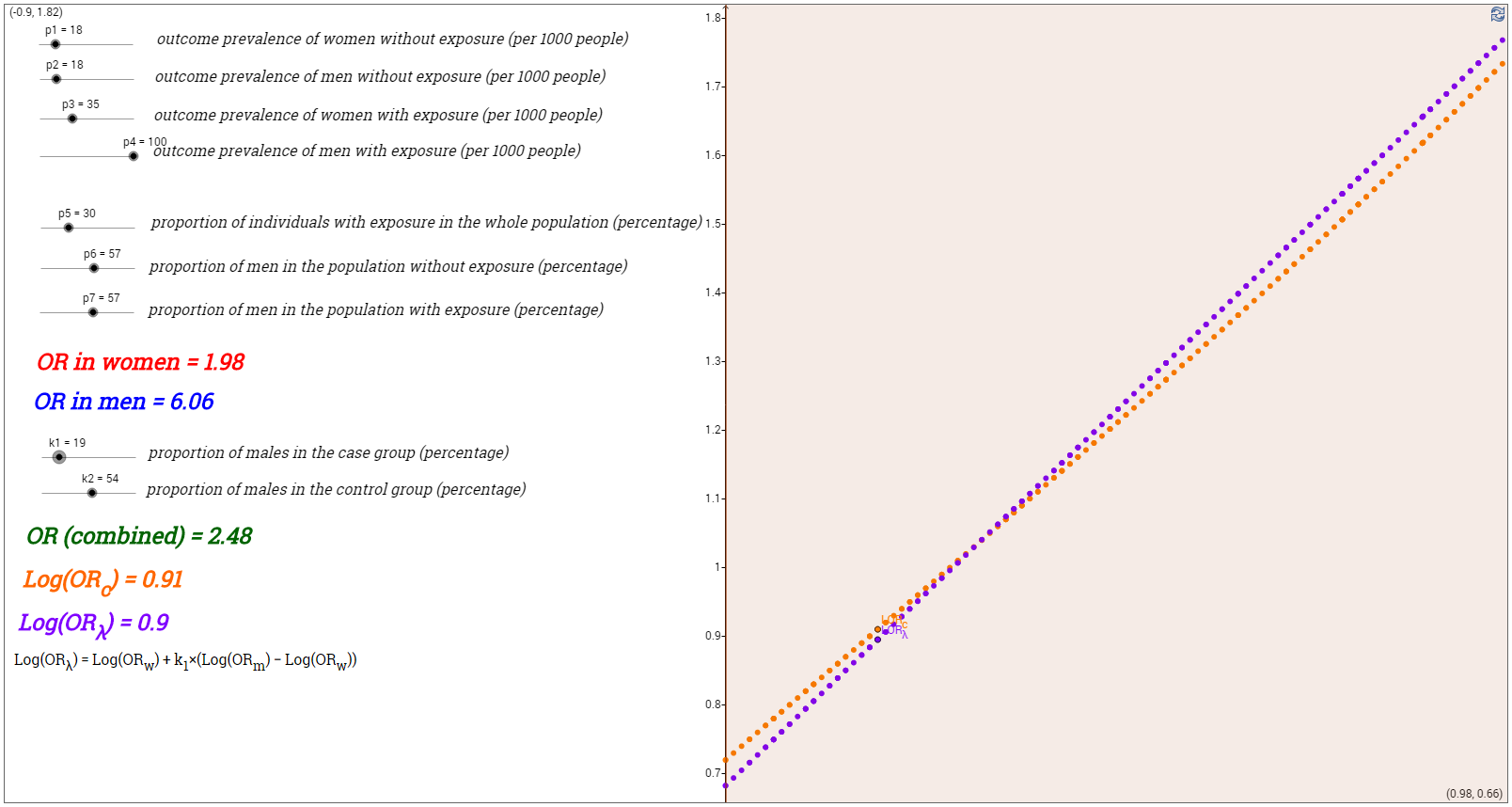


**Figure S2 The screenshot of website when rare assumption was violated.**

The x-axis is the proportion of males in the case group (k1), and the y-axis is the log of odds ratios [including log(ORc) (orange) and log(ORλ)) (purple)].

We could observe the orange trace is not close to purple trace when *p*_1_, *p*_2_, *p*_3_ and *p*_4_ are not rare compared with Figure S1.


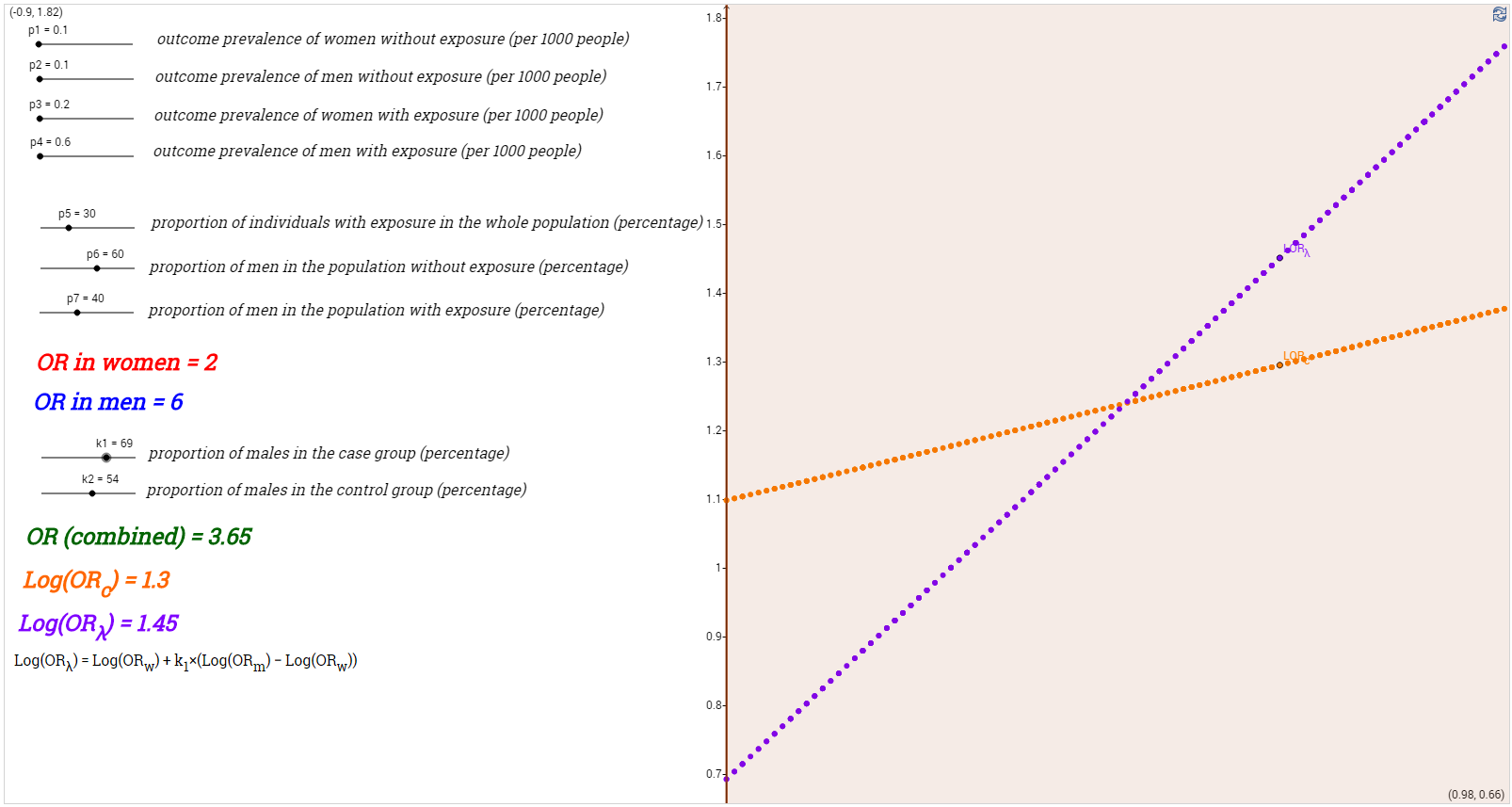


**Figure S3 The screenshot of website when independence assumption was violated.**

The x-axis is the proportion of males in the case group (k1), and the y-axis is the log of odds ratios [including log(ORc) (orange) and log(ORλ)) (purple)].

We could observe the distance between orange trace and purple trace was far when *p*_6_ ≠ *p*_7_.
