# Supplementary material for: Gene-Gene and Gene-Environment Interactions in Meta-Analysis of Genetic Association Studies
Source: PLoS One. 2015 Apr 29;10(4):e0124967. doi: 10.1371/journal.pone.0124967 (PMC4414456; doi:10.1371/journal.pone.0124967)
Supplement: S7 Text — (DOCX) [file pone.0124967.s007.docx]

**The relationship between *G*_1_, *G*_2_, *G*_3_, *G*_4_ and *P*_1_, *P*_2_, *P*_3_, *P*_4_, *P*_5_, *P*_6_, *P*_7_, *P*_8_, *P*_9_, *q*_0_, *q*_1_, *q*_2_.**

$P_{1}=p\left(D=1|x_{1}=0\cap x_{2}=0\right)$; $P_{1}^{'}=1-P_{1}$

$P_{2}=p\left(D=1|x_{1}=0\cap x_{2}=1\right)$; $P_{2}^{'}=1-P_{2}$

$P_{3}=p\left(D=1|x_{1}=1\cap x_{2}=0\right)$; $P_{3}^{'}=1-P_{3}$

$P_{4}=p\left(D=1|x_{1}=1\cap x_{2}=1\right)$; $P_{4}^{'}=1-P_{4}$

$P_{5}=p\left(D=1|x_{1}=2\cap x_{2}=0\right)$; $P_{5}^{'}=1-P_{5}$

$P_{6}=p\left(D=1|x_{1}=2\cap x_{2}=1\right)$; $P_{6}^{'}=1-P_{6}$

$P_{7}=p\left(x_{2}=1|x_{1}=0\right)$; $P_{7}^{'}=1-P_{7}$

$P_{8}=p\left(x_{2}=1|x_{1}=1\right)$; $P_{8}^{'}=1-P_{8}$

$P_{9}=p\left(x_{2}=1|x_{1}=2\right)$; $P_{9}^{'}=1-P_{9}$

$q_{0}=p\left(x_{1}=0\right)=\left(1-\pi_{i}\right)^{2}$; $q_{0}^{'}=1-q_{0}$

$q_{1}=p\left(x_{1}=1\right)=2\pi_{i}\left(1-\pi_{i}\right)$; $q_{1}^{'}=1-q_{1}$

$q_{2}=p\left(x_{1}=2\right)=\pi_{i}^{2}$; $q_{2}^{'}=1-q_{2}$

*D* = disease status (0, health people; 1, patients)

*x*_1_ = SNP (0, homozygous major; 1, heterozygous; 2, homozygous minor)

*x*_2_ = moderator (0, without; 1, with)

π_i_ = minor allele frequency in *i*th study (it was generated at random based on the Blading-Nichols model).

*G*_1_:

$$p\left(x_{1}=0|D=1\cap x_{2}=0\right)=\frac{q_{0}P_{7}^{'}P_{2}}{q_{0}P_{7}^{'}P_{2}+q_{1}P_{8}^{'}P_{4}+q_{2}P_{9}^{'}P_{6}}$$

$$p\left(x_{1}=1|D=1\cap x_{2}=0\right)=\frac{q_{1}P_{8}^{'}P_{4}}{q_{0}P_{7}^{'}P_{2}+q_{1}P_{8}^{'}P_{4}+q_{2}P_{9}^{'}P_{6}}$$

$$p\left(x_{1}=2|D=1\cap x_{2}=0\right)=\frac{q_{2}P_{9}^{'}P_{6}}{q_{0}P_{7}^{'}P_{2}+q_{1}P_{8}^{'}P_{4}+q_{2}P_{9}^{'}P_{6}}$$

*G*_2_:

$$p\left(x_{1}=0|D=1\cap x_{2}=1\right)=\frac{q_{0}P_{7}P_{1}}{q_{0}P_{7}P_{1}+q_{1}P_{8}P_{3}+q_{2}P_{9}P_{5}}$$

$$p\left(x_{1}=1|D=1\cap x_{2}=1\right)=\frac{q_{1}P_{8}P_{3}}{q_{0}P_{7}P_{1}+q_{1}P_{8}P_{3}+q_{2}P_{9}P_{5}}$$

$$p\left(x_{1}=2|D=1\cap x_{2}=1\right)=\frac{q_{2}P_{9}P_{5}}{q_{0}P_{7}P_{1}+q_{1}P_{8}P_{3}+q_{2}P_{9}P_{5}}$$

*G*_3_:

$$p\left(x_{1}=0|D=0\cap x_{2}=0\right)=\frac{q_{0}P_{7}^{'}P_{2}^{'}}{q_{0}P_{7}^{'}P_{2}^{'}+q_{1}P_{8}^{'}P_{4}^{'}+q_{2}P_{9}^{'}P_{6}^{'}}$$

$$p\left(x_{1}=1|D=0\cap x_{2}=0\right)=\frac{q_{1}P_{8}^{'}P_{4}^{'}}{q_{0}P_{7}^{'}P_{2}^{'}+q_{1}P_{8}^{'}P_{4}^{'}+q_{2}P_{9}^{'}P_{6}^{'}}$$

$$p\left(x_{1}=2|D=0\cap x_{2}=0\right)=\frac{q_{2}P_{9}^{'}P_{6}^{'}}{q_{0}P_{7}^{'}P_{2}^{'}+q_{1}P_{8}^{'}P_{4}^{'}+q_{2}P_{9}^{'}P_{6}^{'}}$$

*G*_4_:

$$p\left(x_{1}=0|D=0\cap x_{2}=1\right)=\frac{q_{0}P_{7}P_{1}^{'}}{q_{0}P_{7}P_{1}^{'}+q_{1}P_{8}P_{3}^{'}+q_{2}P_{9}P_{5}^{'}}$$

$$p\left(x_{1}=1|D=0\cap x_{2}=1\right)=\frac{q_{1}P_{8}P_{3}^{'}}{q_{0}P_{7}P_{1}^{'}+q_{1}P_{8}P_{3}^{'}+q_{2}P_{9}P_{5}^{'}}$$

$$p\left(x_{1}=2|D=0\cap x_{2}=1\right)=\frac{q_{2}P_{9}P_{5}^{'}}{q_{0}P_{7}P_{1}^{'}+q_{1}P_{8}P_{3}^{'}+q_{2}P_{9}P_{5}^{'}}$$
