# Supplementary material for: Gene-Gene and Gene-Environment Interactions in Meta-Analysis of Genetic Association Studies
Source: PLoS One. 2015 Apr 29;10(4):e0124967. doi: 10.1371/journal.pone.0124967 (PMC4414456; doi:10.1371/journal.pone.0124967)
Supplement: S1 Table — (DOCX) [file pone.0124967.s008.docx]

**Table S1 Detailed data in the real dataset**

| Study | Case | Control | Proportion of case with moderator | Proportion of control with moderator |  | Study | Case | Control | Proportion of case with moderator | Proportion of control with moderator | |
| --- | --- | --- | --- | --- | --- | --- | --- | --- | --- | --- | --- |
| 1 | 50 | 40 | 70.0% | 55.0% |  | 36 | 110 | 196 | 48.2% | 44.8% |  |
| 2 | 55 | 44 | 70.9% | 52.3% |  | 37 | 172 | 136 | 61.1% | 51.5% |  |
| 3 | 53 | 46 | 64.1% | 52.2% |  | 38 | 59 | 251 | 59.3% | 57.4% |  |
| 4 | 46 | 56 | 41.3% | 39.3% |  | 39 | 158 | 158 | 66.5% | 66.5% |  |
| 5 | 19 | 85 | 50.0% | 61.8% |  | 40 | 121 | 197 | 49.2% | 44.7% |  |
| 6 | 86 | 23 | 66.3% | 65.2% |  | 41 | 145 | 179 | 55.9% | 43.4% |  |
| 7 | 56 | 54 | 33.9% | 37.0% |  | 42 | 160 | 169 | 60.6% | 62.1% |  |
| 8 | 71 | 41 | 54.9% | 48.8% |  | 43 | 157 | 172 | 58.0% | 48.8% |  |
| 9 | 58 | 66 | 54.2% | 53.0% |  | 44 | 133 | 200 | 41.0% | 38.0% |  |
| 10 | 62 | 62 | 59.7% | 48.4% |  | 45 | 179 | 157 | 48.6% | 43.3% |  |
| 11 | 63 | 66 | 47.6% | 37.9% |  | 46 | 174 | 175 | 70.1% | 76.0% |  |
| 12 | 79 | 57 | 53.2% | 56.6% |  | 47 | 190 | 190 | 54.7% | 39.5% |  |
| 13 | 90 | 51 | 43.3% | 33.3% |  | 48 | 117 | 270 | 55.5% | 52.2% |  |
| 14 | 101 | 41 | 64.4% | 58.5% |  | 49 | 198 | 190 | 61.1% | 62.1% |  |
| 15 | 48 | 103 | 43.8% | 49.5% |  | 50 | 242 | 166 | 54.5% | 47.6% |  |
| 16 | 86 | 69 | 59.3% | 59.4% |  | 51 | 208 | 208 | 61.9% | 56.7% |  |
| 17 | 50 | 115 | 66.0% | 42.6% |  | 52 | 196 | 225 | 33.2% | 33.8% |  |
| 18 | 77 | 89 | 48.0% | 39.0% |  | 53 | 235 | 200 | 47.7% | 35.0% |  |
| 19 | 103 | 88 | 58.1% | 56.8% |  | 54 | 291 | 167 | 61.2% | 57.5% |  |
| 20 | 50 | 150 | 46.0% | 50.8% |  | 55 | 168 | 296 | 53.6% | 50.7% |  |
| 21 | 98 | 103 | 33.7% | 49.5% |  | 56 | 337 | 157 | 57.3% | 53.5% |  |
| 22 | 106 | 95 | 57.5% | 51.6% |  | 57 | 240 | 255 | 66.2% | 47.0% |  |
| 23 | 102 | 100 | 52.9% | 30.0% |  | 58 | 260 | 260 | 56.9% | 56.1% |  |
| 24 | 140 | 72 | 40.0% | 31.9% |  | 59 | 261 | 300 | 57.1% | 47.3% |  |
| 25 | 118 | 94 | 47.5% | 44.7% |  | 60 | 260 | 327 | 56.9% | 56.9% |  |
| 26 | 118 | 98 | 72.9% | 73.5% |  | 61 | 311 | 347 | 50.8% | 54.5% |  |
| 27 | 117 | 118 | 53.0% | 54.2% |  | 62 | 295 | 369 | 54.2% | 54.4% |  |
| 28 | 116 | 123 | 41.1% | 26.7% |  | 63 | 180 | 569 | 87.5% | 85.2% |  |
| 29 | 175 | 81 | 38.3% | 45.1% |  | 64 | 515 | 402 | 46.4% | 45.5% |  |
| 30 | 69 | 188 | 59.4% | 58.0% |  | 65 | 453 | 472 | 60.7% | 43.0% |  |
| 31 | 86 | 172 | 77.9% | 71.5% |  | 66 | 745 | 407 | 62.9% | 52.1% |  |
| 32 | 84 | 200 | 31.0% | 33.0% |  | 67 | 748 | 520 | 55.7% | 56.5% |  |
| 33 | 117 | 171 | 66.7% | 50.9% |  | 68 | 461 | 1307 | 53.7% | 43.5% |  |
| 34 | 164 | 124 | 50.6% | 40.3% |  | 69 | 3139 | 605 | 72.8% | 48.3% |  |
| 35 | 161 | 129 | 59.1% | 61.2% |  |  |  |  |  |  |  |
